# The Lunar Display in Colorado

**Published:** 1881-06

**Authors:** 


### THE LUNAR DISPLAY IN COLORADO.

Western papers have given full accounts of the extraordinary lunar phenomena of February 14. A correspondent of the *Illustrated Christian Weekly* describes it vividly thus:

"The evening of February 14 found the thermometer at nineteen below zero. The fire companies of Denver brought out their engines in early evening to extinguish a fire that was raging at the eastern verge of the city, only to find the conflagration down across the plains—the fire of a glorified full moon. For hours thereafter thousands of people forgot the keen, still air in watching a panorama of meteoric marvels spread in the sky.

"Mock moons stood sentinel by the queen of night, two on either side, more intense in color than the moon itself. After a little each of these was surmounted by a brilliant bow. These bows faded, to gradually give place to double halos of lovely violet tint.

"By the time the moon had reached two-thirds the way to the zenith, these phenomena were all gone, and another appeared of far greater brilliancy and magnificence. Faint bands of pink stretched horizontally from the moon to two mock moons in whose huge blazing fires played the prismatic tints of the first rainbow. Then a distinct band of pink outlined a circle round the zenith which enclosed one-fourth of the visible heavens; the narrow band passing directly through the centre of the moon itself and three other moons, a faint one at the farthest western point on the circle, and two others at equal distances from these, the circle being thus intersected by four moons and two slanting prisms on either side of the actual luminary. From these prisms an occasional loop of faint light drooped to the edge of the horizon.

"In the center of the large circle about the zenith, in the very holy of holies, hung a small crescent in the seven colors—a perfect lunar rainbow.

"So impressive a scene has rarely been painted on the always wonderful heavens. And though we may calmly study it and recognize only physical cause and effect—though savants may analyze laws of optics and meteorology, and put this phase here and the other there, is it not all the more surely and beautifully true of the Creator and Lawgiver, that the firmament showeth his handiwork?

